# Evaluation of cell death-inducing activity of *Monilinia* spp. effectors in several plants using a modified TRV expression system

**DOI:** 10.3389/fpls.2024.1428613

**Published:** 2024-08-16

**Authors:** Anselmo López, Jan A. L. van Kan, Henriek G. Beenen, Ramon Dolcet-Sanjuan, Neus Teixidó, Rosario Torres, Laura Vilanova

**Affiliations:** ^1^ IRTA, Postharvest Programme, Edifici Fruitcentre, Parc Agrobiotech Lleida, Lleida, Catalonia, Spain; ^2^ Laboratory of Phytopathology, Wageningen University, Wageningen, Netherlands; ^3^ IRTA, Plant In Vitro Culture Laboratory, Fruticulture Program, Parc Agrobiotech Lleida, Lleida, Catalonia, Spain

**Keywords:** *Monilinia fructicola*, *Prunus* spp., transient expression, NLPs, necrotrophic fungi

## Abstract

**Introduction:**

Brown rot is the most important fungal disease affecting stone fruit and it is mainly caused by *Monilinia fructicola*, *M. laxa* and *M. fructigena*. *Monilinia* spp. are necrotrophic plant pathogens with the ability to induce plant cell death by the secretion of different phytotoxic molecules, including proteins or metabolites that are collectively referred to as necrotrophic effectors (NEs).

**Methods:**

We exploited the genomes of *M. fructicola*, *M. laxa* and *M. fructigena* to identify their common group of secreted effector proteins and tested the ability of a selected set of effectors to induce cell death in *Nicotiana benthamiana*, *Solanum lycopersicum* and *Prunus* spp. leaves.

**Results:**

Fourteen candidate effector genes of *M. fructicola*, which displayed high expression during infection, were transiently expressed in plants by agroinfiltration using a modified *Tobacco Rattle Virus* (TRV)-based expression system. Some, but not all, effectors triggered leaf discoloration or cell death in *N. benthamiana* and *S. lycopersicum*, which are non-hosts for *Monilinia* and in *Prunus* spp., which are the natural hosts. The effector MFRU_030g00190 induced cell death in almost all *Prunus* genotypes tested, but not in the Solanaceous plants, while MFRU_014g02060, which is an ortholog to BcNep1, caused necrosis in all plant species tested.

**Conclusion:**

This method provides opportunities for screening *Prunus* germplasm with *Monilinia* effector proteins, to serve as a tool for identifying genetic loci that confer susceptibility to brown rot disease.

## 1 Introduction

One of the most important fungal diseases affecting stone fruit is brown rot. It causes large economic losses, principally in peaches and nectarines, but also affects other stone fruit like cherries, apricots, and almonds (Ollero-Lara et al., 2019; Baró-Montel et al., 2019a). The infection occurs in the field, starting from overwintered mummies that generate the primary inoculum for emerging flowers and fruit during spring and summer (Villarino et al., 2010). Nevertheless, the main economic losses of brown rot appear during the postharvest handling, storage, and transportation (Casals et al., 2022).

The genus *Monilinia* are Ascomycetes included in the *Sclerotiniaceae* family and it contains 37 species, most of which are plant pathogens (Index Fungorum, 2023). The three species mainly responsible for stone fruit brown rot are *Monilinia fructicola*, *M. laxa* and *M. fructigena*. Out of these species, *M. fructicola* is considered to cause the most severe losses in stone fruit (Villarino et al., 2013). Occurrence and distribution of the three species has traditionally differed worldwide. While *M*. *laxa* and *M. fructigena* have long been the main causal agent of brown rot in stone fruit in Europe, *M. fructicola* has traditionally affected stone fruit in India, Japan, Oceania and the Americas. *M. fructicola* was first detected in Europe in fruit orchards in France in 2001 (Lichou et al., 2002) and it has since migrated into several European countries (Hrustić et al., 2015). In Spain it was first detected in Ebro Valley orchards in 2009 (De Cal et al., 2009).

Depending on the strategies developed to interact with the host, plant pathogenic fungi are classified as having either a necrotrophic, hemibiotrophic or biotrophic lifestyle (Tan et al., 2010). In a classical biotrophic lifestyle, the interaction between an effector (acting as an avirulence gene) and its specific plant receptor (encoded by an *R*-gene) leads to a defense response causing host cell death and stopping the infection process (Tan et al., 2015). By contrast, necrotrophic pathogens secrete cell death-inducing effectors with the capacity to trigger host programmed cell death (PCD), which is a key step in successful plant colonization and disease development (Tan et al., 2010; Kabbage et al., 2017; Faris and Friesen, 2020; Malvestiti and van Kan, in press).

Cell death-inducing effectors can either be secondary metabolites or relatively small proteins (Tan et al., 2010). Depending on their target in host plants, the effectors can be grouped as apoplastic or cytoplasmic effectors. Both are delivered into the plant, however, apoplastic effectors are secreted into the plant apoplast, where they interact with extracellular receptors, while cytoplasmic effectors are translocated inside the plant cell (Selin et al., 2016). In recent years, many studies have been devoted to host cell death-inducing effectors produced by necrotrophic pathogens affecting wheat, barley, and maize with the *Parastagonospora nodorum*-wheat pathosystem serving as a model for the interaction between necrotrophic fungi and their host (Friesen and Faris, 2021; Shao et al., 2021). Three fungal effector genes have been cloned along with their target genes in wheat: Tox1-*Snn1*, Tox3-*Snn3* and ToxA-*Tsn1* (Friesen and Faris, 2021). Three more effector genes (Tox4, Tox5 and Tox 267) have been cloned from *P. nodorum* and five wheat loci involved in recognition of these effectors were genetically mapped (reviewed in Malvestiti and van Kan, in press).

The best characterized necrotrophic pathogen from the Sclerotiniaceae family is *Botrytis cinerea*. A large number of cell death-inducing effectors have been studied and functionally characterized (Leisen et al., 2022; Bi et al., 2023). The availability of high-quality annotations of different *Monilinia* spp. genomes enables studies on effector proteins of these species (Landi et al., 2018; Naranjo-Ortíz et al., 2018; Rivera et al., 2018; De Miccolis Angelini et al., 2019; Marcet-Houben et al., 2021). Genome comparison among five *Monilinia* spp. highlighted the low variability in the CAZome, some common secondary metabolism clusters, and several potential candidate effector genes (Marcet-Houben et al., 2021). Despite the available genome information, molecular-genetic studies on the interaction between *Monilinia* spp. and *Prunus* spp. are in their infancy as compared to other Sclerotiniaceae. To investigate the role of *Monilinia* effectors in the infection process, it is desirable to have a tool to test their cell death-inducing capacity in *Prunus* spp. tissues. Previous studies in *B. cinerea* used heterologous expression in *Pichia pastoris* to produce a set of endopolygalacturonases (Kars et al., 2005), however, that is a time-consuming technique with relatively low efficiency. Functional screens with effector proteins of biotrophic pathogens often make use of transient expression systems using plant virus vectors as expression platform (Nasir et al., 2005; Vleeshouwers and Oliver, 2014). Potato Virus X (PVX) is often used for these purposes when screening germplasm of Solanaceae (Domazakis et al., 2017), however this virus was not reported to replicate in *Prunus* spp. *Tobacco Rattle Virus* (TRV), however, can replicate in a wide range of plant species, including *Prunus* spp. leaves (Bai et al., 2016) and this virus was considered to be potentially useful for expressing *M. fructicola* effector proteins in *Prunus* spp. leaves.

The aim of this study was to identify the common effectorome from the three most important species of *Monilinia* in stone fruit, *M. fructicola*, *M. laxa* and *M. fructigena*, and to study the cell death-inducing capacity of a subset of *M. fructicola* effectors using modified TRV constructs for expressing them in leaves of non-hosts and hosts of *Monilinia* spp.

## 2 Materials and methods

### 2.1 Identification of the common effectorome from three *Monilinia* species

The genomes of *M. fructigena* strain gena6 (BioProject Code PRJNA707424), *M. laxa* strain 8L (BioProject PRJNA433296; Naranjo-Ortíz et al., 2018) and *M. fructicola* strain CPMC6 (BioProject PRJNA503180; Vilanova et al., 2021) as well as the comparative genomic analysis (Marcet-Houben et al., 2021) were used for the identification of the effector genes for each species. Specifically, we used the effectorome data from Vilanova et al. (2021) for *M. fructicola* and the effectorome data from Marcet-Houben et al. (2021) for *M. laxa* and *M*. *fructigena.*

Each predicted effector gene from all three species was blasted to the other species one to one, using Genome Workbench and PhylomeDB (E-value cut-off 0.001 and query coverage ≥ 80%). The bidirectional best blast hits were listed and used for further analyses (**Supplementary** **Table S1**).

Transcriptionally inactive genes were eliminated based on RNA-Seq data from *M. laxa-* infected fruits (Balsells-Llauradó et al., 2020) and *M. fructicola-*infected leaves (Vilanova et al., unpublished).

### 2.2 Plant material and fungal culture

In this study, leaves from different plant species were used: *Nicotiana benthamiana*, *Solanum lycopersicum*, and *Prunus* spp. *N. benthamiana* and *S. lycopersicum* served as model plants to ensure the TRV system is well established. Additionally, *N. benthamiana* was used to detect non-host specific effectors while *S. lycopersicum* and *Prunus* spp. were used to identify host-specific effectors, with a particular focus on *Prunus* spp. as it is the actual host of *Monilinia* spp.

*N. benthamiana* plants were grown in a climate chamber located at IRTA Fruitcentre in Lleida at 25°C under a 16 h light/8 h dark cycle, *Solanum lycopersicum* var. Moneymaker plants were grown in the greenhouses located at University of Lleida. Furthermore, *Prunus* spp. were included due to their role as the pathogen host. Initially, *Prunus persica* plants derived from directed crosses were employed to assess the viability of agroinfiltration in this plant species. Subsequently, five well-characterized genotypes were selected for further investigation. These individuals included: *Cadaman* (*Prunus davidiana × Prunus persica*), *Prunus davidiana* and three different genotypes denotated 37p15-13, 37p15-16, and 37p15-17. These three genotypes are a BC1 population derived from a cross between the peach cultivar *Nectatop* and *Cadaman*. This population has been previously generated and genotyped (Zaracho-Echagüe et al., 2022). All *Prunus* spp. plants were *in vitro* clonally propagated as described (Cantabella et al., 2021). Briefly, *in vitro* shoot tip cultures were established from field grown plants, propagated by axillary branching in MS (Murashige & Skoog) medium supplemented with 5 μM benzylaminopurine (BAP). Shoot elongation was achieved adding liquid MS media, without hormones, to the semisolid multiplication medium. Root induction on *in vitro* elongated shoots was conducted in half-strength MS media supplemented with 10 μM indole-3-acetic acid (IAA), followed by transfer to auxin-free medium amended with vermiculite, and afterwards acclimated to greenhouse conditions.

The *M. fructicola* strain CPMC6 belongs to the collection of the Postharvest Pathology group of IRTA (Lleida, Spain) and is deposited in the Spanish Culture Type Collection (CECT 21105). For long-term storage, the isolate was kept as a conidial suspension in 20% glycerol at -80°C. Cultures were grown in malt extract plates (MEA) and incubated at 25°C in darkness for 10-12 days before use.

### 2.3 RNA isolation and cDNA synthesis

*M. fructicola* was grown on cellophane on MEA incubated at 24°C for 10-12 days, and RNA was extracted from freeze-dried mycelium using RNeasy^®^ Plant Mini Kit (Qiagen, Hilden, Germany) according to manufacturer´s recommendations. RNA from *M. fructicola* decayed stone fruit tissue was extracted following the methodology described by Baró-Montel et al. (2019c). For elimination of genomic DNA, RNA was treated with TURBO™ DNase (Invitrogen, ThermoFisher Scientific, Carlsbad, CA, USA). First strand cDNA was synthesized using the SuperScript™ IV First-Strand Synthesis Kit (Invitrogen, ThermoFisher Scientific, Carlsbad, CA, USA) and used for gene amplification.

### 2.4 Amplification of candidate effector genes

All oligonucleotides used are listed in **Supplementary Table S2**. Primers were designed using the NEBuilder Assembly Tool (New England Biolabs, MA, USA) to amplify two fragments for each selected gene. The coding sequence of the signal peptide of the pathogenesis-related protein from *N. benthamiana* (PR1) and the coding sequence (CDS) of the mature candidate effector excluding the N-terminal signal peptide were amplified. Fragments for MFRU002g05260 and MFRU030g00580 genes were amplified directly from the plasmids obtained by Vilanova et al. (2021). Fragment amplification for cloning was performed in a volume of 50 µL containing 1µM of both forward and reverse primers, 0.2 mM of each dNTP, 1X PFU buffer (Promega, Leiden, The Netherlands), 1.5 U PFU polymerase (Promega), and 2 µL of template (100 ng of cDNA from *M. fructicola* or 100 ng of plasmid). PCR program for both fragments of MFRU004g02710, MFRU004g02720, MFRU014g02060, MFRU048g00370, MFRU004g00620 and MFRU005g00910 was 95°C for 2 min; 10 cycles of 95°C for 30 s, 55°C for 30 s and 72°C for 1 min and 25 cycles with an annealing temperature of 60°C instead of 55°C, ending with a final step at 72°C for 5 min. PCR program for the PR1 fragment of MFRU004g02090, MFRU008g03430, MFRU010g02580, MFRU018g01470, MFRU027g00340 and MFRU030g00190 was 95°C for 2 min; 35 cycles of 95°C for 15 s, 55°C for 30 s, and 72°C for 30 s, ending with a final step at 72°C for 5 min. PCR program for the other fragments was 95°C for 2 min; 35 cycles of 95°C for 15 s, 52°C for 30 s, and 72°C for 80 s, ending with a final step at 72°C for 5 min. PCR products were visualized in a range of 1-3% agarose gel.

### 2.5 Vector construction and cloning

pTRV2 VIGS vector pYL156 (Liu et al., 2002) (kindly provided by Laurens Deurhof, Wageningen University) was modified by removing remainders of the TRV2b protein coding sequence and inserting a sequence encoding a plant secretion signal peptide (derived from the tobacco pathogenesis-related protein PR1a), joint in frame to the coding sequence of a mature *Monilinia* effector gene, lacking the fungal secretion signal peptide sequence. Constructs were made using the NEBuilder^®^ HiFi DNA Assembly Cloning kit (New England BioLabs, MA, USA). The pTRV2 vector was linearized using restriction enzymes (New England BioLabs, MA, USA), either PvuII, EcoRI, AatII or BamHI (New England BioLabs, MA, USA) at 37°C, according to manufacturer’s recommendations. Each digestion was done one by one followed by a purification step. Fragments and the linearized vector were incubated with Clonexpress II one step cloning kit (Vazyme, Nanjing, China) or NEBuilder HiFi DNA Assembly Master Mix (New England BioLabs, MA, USA) following manufacturer´s recommendations (**Supplementary Figure S1**). From 10 ng to 1000 ng of ligated plasmid was transformed into ultra-competent DH5α *E. coli* cells. Cells were selected on LB medium with kanamycin and incubated overnight at 37°C. Plasmids were isolated using the QIAprep Spin Miniprep kit (Qiagen, Hilden, Germany) according to manufacturer’s recommendations. To check if the selected genes were integrated in the pTRV2 plasmids, PCR was performed using IBIAN^®^-Taq DNA Polymerase (Ibian Technologies, Zaragoza, Spain) in a total volume of 25 µL containing 0.5 µM of both forward and reverse primer, 0.2 mM of each dNTP, 1X IBIAN Buffer, 1.25 U IBIAN-Taq^®^ DNA polymerase and 0.5 uL of the pTRV2 vector. PCR program was 94°C for 2 min; 30 cycles of 94°C for 10 s, 55°C for 20 s and 72°C for 1,5 min, ending with a final step of 72°C for 5 min. Those clones with similar size to the expected were sequenced for further confirmation.

### 2.6 *Agrobacterium tumefaciens* mediated-transient expression in *Nicotiana benthamiana*, *Solanum lycopersicum* and *Prunus* spp.

Fifty ng of plasmid were added to 50 μL of electro-competent *Agrobacterium tumefaciens* cells (strain GV3101). After electroporation, cells were plated in LB petri dishes supplemented with kanamycin and rifampicin and incubated at 28°C for two days. *Agrobacterium* cultures were transferred into 15 mL YEB medium containing 20 µM acetosyringone, 50 mg/L kanamycin and 20 mg/L rifampicin and they were grown during 22 h at 28°C and 200 rpm. Pellet was resuspended in MMAi (1 mM MES, 10 mM MgCl_2_, 200 µM acetosyringone) buffer at OD600 = 1.6. Each solution of the different pTRV2 strains were mixed 1:1 with pTRV1 (Liu et al., 2002) and incubated during 1 h at room temperature. The different mixes were infiltrated using a needleless 1 mL syringe in leaves of 4-6 weeks old *N. benthamiana* plants, 5-6 weeks old *S. lycopersicum* and in *Prunus* spp. plants with at least 10 leaves. Pictures of responses in all *N*. *benthamiana* and *S. lycopersicum* plants were taken 5 and 7 days postinfiltration, and for *Prunus* spp. leaves, the symptoms were observed 10 days postinfiltration. This experiment was replicated three times, and each *Agrobacterium* suspension was infiltrated in three different leaves from three different plants.

## 3 Results

### 3.1 Identification of the common effectorome from three *Monilinia* spp.

**Figure 1:**
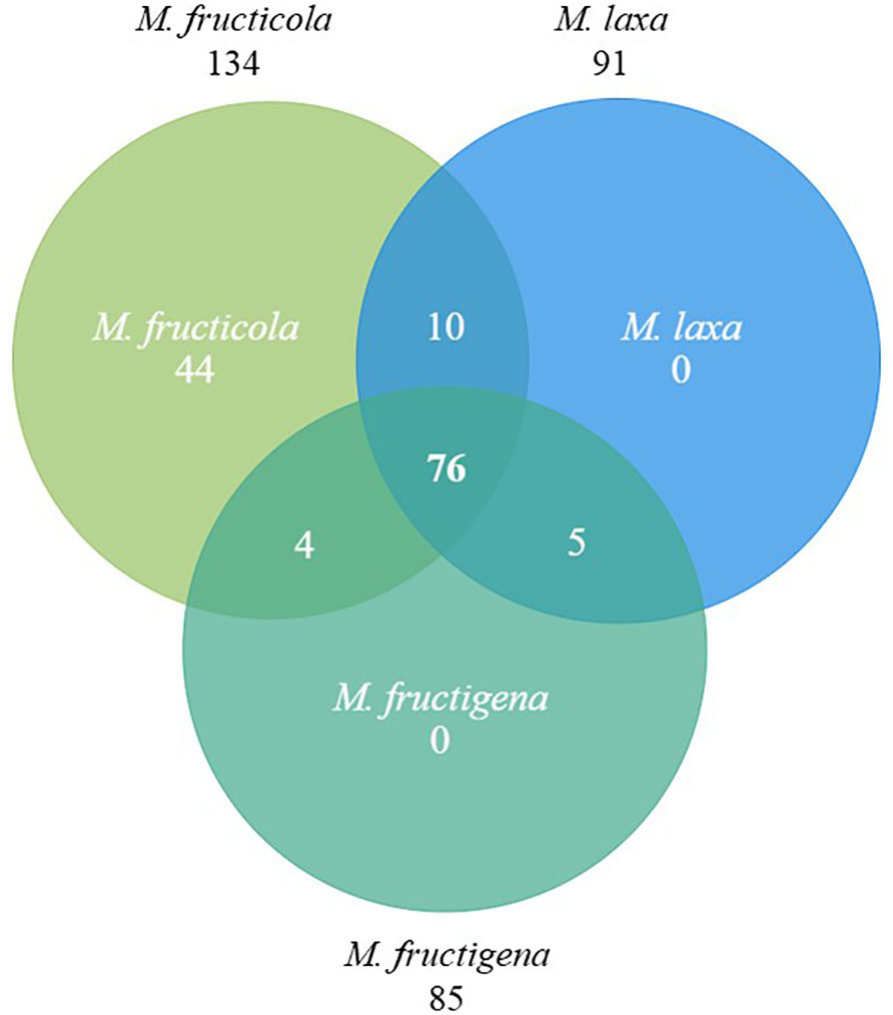
Venn diagram showing the effector genes shared among *Monilinia fructicola, M. laxa* and *M. fructigena*. Each circle represents the set of effectors for each species, with overlapping areas indicating shared effector genes between species.

The identification of the common effector genes shared between the three main *Monilinia* spp. was based on the analysis of Marcet-Houben et al. (2021). Bidirectional blast analysis was performed between effector genes in *M. fructicola*, *M. laxa* and *M. fructigena* in all possible combinations resulting in 76 effector genes shared among the three *Monilinia* spp. (**Figure 1**; **Supplementary Tables S3**, **S4**).

For these 76 common effectors, information about their expression during the infection of *M. fructicola* in leaves (Vilanova et al., 2021) and of *M. laxa* in nectarines (Balsells-Llauradó et al., 2020) was used to eventually select 14 effector genes for transient expression analysis of cell death-induction in different plant species (**Table 1**).

**Table 1:** *Monilinia fructicola* effector genes tested for cell death-induction by *Agrobacterium tumefaciens* transient expression (ATTA) in plants.

Protein	Ortholog in *M. laxa*	Ortholog in *M. fructigena*	Protein Size (aa)	#Cys	Description
MFRU_002g05260	Monilinia_093480	g5377	221	10	CFEM domain
MFRU_004g00620	Monilinia_063060	g1907	216	4	Hypothetical protein
MFRU_004g02090	Monilinia_028260	g4487	161	0	Hypothetical protein
MFRU_004g02710	Monilinia_064810	g1289	105	6	Hypothetical protein
MFRU_004g02720	Monilinia_064800	g1290	239	4	Hypothetical protein
MFRU_005g00910	Monilinia_024190	g6968	181	4	Hypothetical protein
MFRU_008g03430	Monilinia_059180	g6370	204	0	Hypothetical protein
MFRU_010g02580	Monilinia_060470	g5946	108	0	Hypothetical protein
MFRU_014g02060	Monilinia_013380	g6703	247	3	NEP1 Like Protein (NLP)
MFRU_018g01470	Monilinia_010100	g5286	342	0	Hypothetical protein
MFRU_027g00340	Monilinia_089850	g5226	273	0	Hypothetical protein
MFRU_030g00190	Monilinia_063010	g8181	126	6	Hypothetical protein
MFRU_030g00580	Monilinia_012000	g3324	160	6	Hypothetical protein
MFRU_048g00370	Monilinia_030790	g971	147	9	Hypothetical protein

### 3.2 Response to transient expression in *Nicotiana benthamiana* and *Solanum lycopersicum*

**Figure 2:**
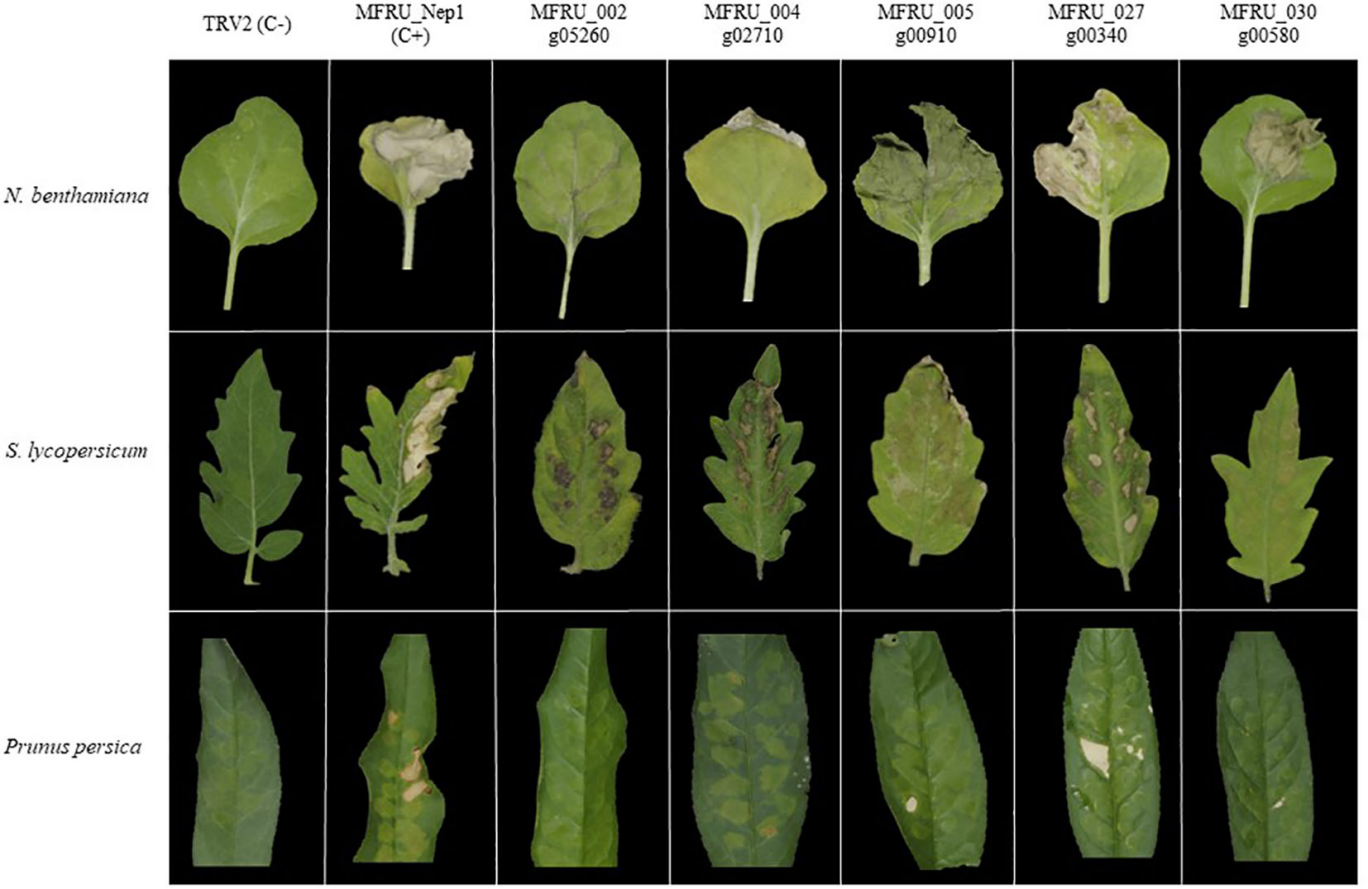
*Agrobacterium tumefaciens* mediated-transient expression (ATTA) using modified TRV binary vector for expressing several *Monilinia fructicola* candidate effector proteins in *N. benthamiana* (top row) and *S. lycopersicum* (middle row) and *Prunus persica* (bottom row). First column is the positive control, and the second column is the negative control. Pictures were taken 7 days postinfiltration.

The cell death-inducing capacity of each of the 14 candidate effector genes from *M. fructicola* was tested by ATTA using a modified binary vector containing the *Tobacco Rattle Virus* (pTRV2 mixed 1:1 with pTRV1) in three different plant species (**Supplementary Figure S2**). MFRU_014g02060 is an ortholog to BcNep1, which was previously shown to have a strong necrotizing activity in *N. benthamiana* leaves (Schouten et al., 2008), and it was used as a positive control. As negative control, we used an empty TRV2 vector combined with TRV1 (**Figure 2**).

In *N. benthamiana* leaves, six of the 14 candidate genes caused necrosis in the infiltrated area at five days postinfiltration (**Table 2**), with the genes MFRU_027g00340, MFRU_030g00580 and MFRU_005g00910 showing the strongest cell death-inducing activity (**Figure 2**).

**Table 2:** Summary of *Agrobacterium* infiltration and necrosis observation in various plant species for *Monilinia fructicola* proteins.

	N. benthamiana	S. lycopersicum	P. persica	Cadaman	P. davidiana	37p15.13	37p15.16	37p15.17
MFRU_002g05260	**-**	**+**	**-**	ND	ND	ND	ND	ND
MFRU_004g00620	**-**	**-**	**-**	ND	ND	ND	ND	ND
MFRU_004g02090	**-**	**-**	**-**	ND	ND	ND	ND	ND
MFRU_004g02710	**+**	**+**	**+**	**-**	**-**	**+**	**-**	**+**
MFRU_004g02720	**-**	**+**	**+**	**-**	ND	**-**	**-**	**-**
MFRU_005g00910	**+**	**+**	**+**	**-**	**-**	**-**	**+**	**-**
MFRU_008g03430	**-**	**-**	**-**	ND	ND	ND	ND	ND
MFRU_010g02580	**-**	**-**	**-**	ND	ND	ND	ND	ND
MFRU_014g02060	**+**	**+**	**+**	**-**	**+**	**+**	**+**	**+**
MFRU_018g01470	**-**	**-**	**-**	ND	ND	ND	ND	ND
MFRU_027g00340	**+**	**+**	**+**	**-**	**-**	**-**	**+**	**-**
MFRU_030g00190	**-**	**-**	**-**	**-**	**+**	**+**	**+**	**+**
MFRU_030g00580	**+**	**-**	**-**	ND	ND	ND	ND	ND
MFRU_048g00370	**+**	**-**	**+**	ND	ND	ND	ND	ND

Symbols “+” and “-” represent the presence or absence of necrosis, respectively. “ND” indicates cases where the experiment was not conducted.

Similar experiments were conducted in *S. lycopersicum* leaves and some differences were observed. Compared to *N. benthamiana*, the same number of candidate genes induced necrosis in the infiltrated area (**Table 2**), but these genes were different. MFRU_014g02060, MFRU_004g02710, MFRU_005g00910 and MFRU_027g00340 induced necrosis in the infiltrated area in both tomato and *N. benthamiana* leaves (**Figure 2**). No necrosis was observed in tomato leaves upon infiltration with the MFRU_030g00580 construct, while this produced significant necrosis in *N. benthamiana*. In contrast, clear necrosis was observed after infiltrating MFRU_002g05260 and MFRU_004g02710 in tomato, whereas no necrosis were noted in *N. benthamiana*. The cell death-inducing capacity of the MFRU_027g00340 construct exhibited the closest response to that of the positive control in both species.

### 3.3 Effect of transient gene expression in *Prunus* spp. leaves

As stone fruit is the real host of *M. fructicola*, the next step was to test the fourteen selected effector genes in host-specific tissues, specifically stone fruit leaves, using the transient TRV-mediated expression system developed in this work.

The constructs for the expression of the fourteen effector genes were tested in leaves of *Prunus persica*, and symptoms became apparent for some constructs 10 days postinfiltration (**Supplementary Figure S2**). Six of the fourteen genes induced necrosis in the infiltrated area and surroundings (**Table 2**). These genes were: MFRU_004g02710, MFRU_004g02720, MFRU_005g00910, MFRU_014g02060 (MFRU_Nep1), MFRU_027g00340, and MFRU_48g00370. In contrast with *N. benthamiana* and tomato, respectively, no necrosis symptoms were observed after the infiltration of MFRU_002g05260 construct and MFRU_030g00580 in *Prunus* leaves. The constructs that were able to induce necrosis symptoms in all three plant species after infiltration were MFRU_004g02710, MFRU_005g0910, MFRU_014g02060 and MFRU_027g00340 constructs (**Table 2**).

**Figure 3:**
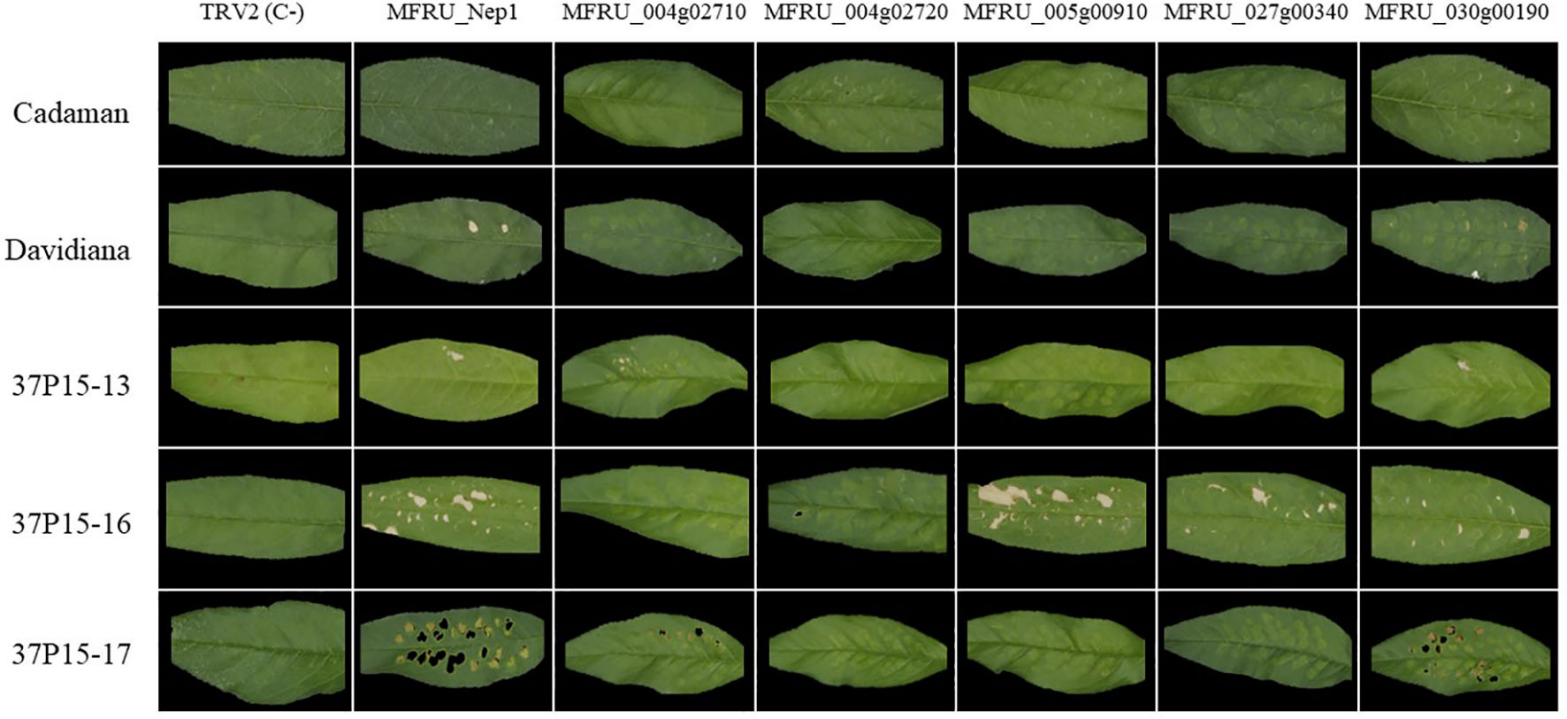
*Agrobacterium* mediated-transient expression (ATTA) using modified TRV construct for expressing several *M. fructicola* candidate effector proteins in different *Prunus* genotypes leaves. Columns contain the different gene constructs and rows contain are the different *Prunus* genotypes. Pictures were taken 10 days postinfiltration.

Subsequently, we tested the cell death-inducing capacity of six candidate effector genes in five different *Prunus* genotypes: Cadaman, *Prunus davidiana* and three genotypes called 37p15-13, 37p15-16, and 37p15-17 (**Figure 3**). Particularly, genotype 37p15-16 showed the most pronounced necrosis after the infiltration of all six candidate effector genes, except for MFRU_004g02710 and MFRU_004g02720, which only caused mild discoloration (**Supplementary Figure S3**, **Table 2**). In this genotype, the gene that produced more necrosis in the infiltrated area and surroundings was MFRU_005g00910, together with MFRU_014g02060 (**Figure 3**). On the other hand, no response was observed after the infiltration of any construct in the hybrid Cadaman. *P. davidiana* was the species in which symptoms of necrosis only appeared after the infiltration of MFRU_014g02060 and MFRU_030g00190. These 2 candidate effector genes, together with MFRU_004g02710, also induced necrosis and cell death in the leaves of the 37P15-17 and 37P15-13 genotypes (**Figure 3**).

## 4 Discussion

*M. fructicola* is considered to be the most economically damaging *Monilinia* spp. in stone fruit in Europe, even more than endemic *Monilinia* spp. such as *M. fructigena* and *M. laxa*. Its ability to produce more conidia than other *Monilinia* spp. may help it succeed as an invasive species (Villarino et al., 2013). In an effort to gain insights into the pathogenic mechanisms of *M. fructicola*, several groups have produced genome data for *Monilinia* spp. in recent years (Naranjo-Ortíz et al., 2018; Rivera et al., 2018; De Miccolis Angelini et al., 2019; Vilanova et al., 2021).

Valero-Jiménez et al. (2019) tried to identify species-specific effectors that were determinant in host specificity, by comparing the effector set of nine different *Botrytis* spp. No host-specific relation was found between the different genes found in the different species and its hosts, and furthermore, all effectors that were previously functionally characterized in *B. cinerea* were also present in the other species analyzed. Taking this into account, the approach of the present study was the opposite, identifying the common effectors shared among three *Monilinia* species responsible for causing brown rot in stone fruit. In the case of *M. fructicola*, the number of effector genes identified by Marcet-Houben et al. (2021) was different when compared to the number of effector genes defined by Vilanova et al. (2021). The numbers of effector genes that we used differ from the study by De Miccolis Angelini et al. (2022) which had fewer effector genes in all three species, and the study by Akhoon et al. (2023), which had significantly more effector genes in all three species. This discrepancy is due to the use of distinct criteria to determine what constitutes an effector and to differences in prediction tools and choices in the stringency of prediction parameters. Moreover, manual curation efforts as conducted by Vilanova et al. (2021) may further contribute to variability in gene numbers and lists. The strategy used in this study for identifying effectors shared between all three *Monilinia* species was based on bidirectional blasts of the effector repertoire of each species to the other species, one by one. After all comparisons, the number of effector genes shared among the three *Monilinia* spp. genomes in this study was 76. The effectors described by De Miccolis Angelini et al. (2022) and Akhoon et al. (2023) that are not represented in our effector lists might be excellent candidates for testing using the TRV-based expression system.

Viral vectors are used in effectoromic screenings, but those studies are mostly done with Potato Virus X on Solanaceae (Nasir et al., 2005; Vleeshouwers and Oliver, 2014). As PVX was not reported to infect *Prunus*, we decided to switch to a virus that is reported to infect and replicate in *Prunus*, namely Tobacco Rattle Virus (TRV). TRV-based expression systems are commonly used in plants for the purpose of achieving virus-induced gene silencing (VIGS). It serves as a high-throughput tool to investigate gene function in several plant species (Hartl et al., 2008). In this study, a TRV vector designed for VIGS (Liu et al., 2002) was modified to enable the transient expression of fungal effector genes in plants. The virus possesses a bipartite genome, with RNA2 encoding two proteins redundant for viral replication but essential for viral transmission by nematodes (Macfarlane, 2010). Liu et al. (2002) crafted a binary vector construct that includes T-DNA borders flanking an expression cassette containing TRV RNA2 sequences and a multiple cloning site for the insertion of desired sequences. *Agrobacterium tumefaciens* carrying this binary vector construct can be mixed with another *A. tumefaciens* containing TRV RNA1 sequences and then infiltrated into plants. The mixture of these *Agrobacterium* strains allows for the transformation of plant cells, facilitating the expression and subsequent replication of both viral RNAs. These RNAs can disperse throughout the host plant, enabling the expression of heterologous genes, particularly fungal effectors in this case.

With this modified virus construct, the cell death-inducing capacity of putative effector genes of *M. fructicola* could be tested in leaf tissues of different plants, including its host, *Prunus.* The development of a high-throughput screening method using a modified TRV expression vector offers a good perspective in disease resistance breeding against plant pathogens. The strategy can be applied to study the response to fungal effector proteins in plants that can be infected by this virus such as strawberry, wheat, maize, and others (Zeng et al., 2019). This methodology provides an alternative strategy to heterologous protein production in bacteria or yeasts, which is more labour- and time-intensive. Besides this, these kind of effector assays could be complemented with infection assays and field trials (Vleeshouwers and Oliver, 2014) to test whether stone fruit, which do not show necrosis when infiltrating by fungal effectors, are less susceptible to *Monilinia* spp. In recent years, several genetic and quantitative genetic studies have revealed QTLs or markers related to brown rot resistance in peach (Baró-Montel et al., 2019b). In the case of *Monilinia* spp., knowledge about the capacity of its effectors to induce cell death and promote virulence can be exploited to genetically map and eventually clone susceptibility genes with the use of genomic tools (Martínez-García et al., 2013; Pacheco et al., 2014).

This study has identified several *M. fructicola* effector genes that induce cell death in its host plants leaf tissue. Necrosis was observed in leaves of almost all *Prunus* spp. tested upon infiltration of MFRU_030g00190, while neither *N. benthamiana* nor tomato responded to this protein. We hypothesize that the recognition of necrotrophic effectors (NEs) secreted by *Monilinia* species by receptor proteins of *Prunus* leads to host cell death and thereby confers susceptibility. If a pathogen does not produce a particular effector, or if the host does not express the susceptibility gene, the pathogen cannot invade the host, resulting in resistance (Faris and Friesen, 2020). This susceptibility mechanism is well-studied in the interaction between wheat and the necrotrophic fungi *Parastagonospora nodorum* and *Pyrenophora tritici*-*repentis* (Tan et al., 2012; Virdi et al., 2016; Faris and Friesen, 2020). Another well-characterized pathosystem involves fungi from the *Cochliobolus* genus, which produce cell death-inducing metabolites that are crucial for pathogenicity. In particular, *C. victoriae* produces victorin, conferring pathogenicity to oat genotypes carrying the *Vb* locus as a susceptibility determinant (reviewed in Malvestiti and van Kan, in press). Within the Sclerotiniaceae family, *Botrytis cinerea* stands out as the most extensively studied necrotrophic pathogen. It secretes several cell wall degrading enzymes (CWDEs) with NE activity, as well as NEs that lack an annotated enzymatic domain (for example BcNep1 and BcNep2), and secondary metabolites that contribute to virulence (Malvestiti and van Kan, in press; Bi et al., 2023).

In contrast with what it was observed with MFRU_030g00190, two genes (MFRU_005g00910 and MFRU_014g02060) were shown to cause necrosis in all three plant species tested. The MFRU_014g02060 protein is the ortholog to NEP1 proteins from *Botrytis* spp. NEP1-like proteins (NLPs) have been identified in plant pathogenic fungi, bacteria, and oomycetes, despite there has been ambiguity regarding their host range. While functionally characterized cytolytic NLPs have traditionally shown broad activity exclusively on dicotyledonous plant species (Seidl and Van den Ackerveken, 2019), recent reports revealed sensitivity of several monocotyledonous plants to NLPs as well (Steentjes et al., 2022). This underscores the importance of further investigating the host range and functional roles of these effectors. To do so, the generation of knockout mutants and even multigenic knockout mutants for the most interesting effectors identified in this study would be a promising approach. As demonstrated in *B. cinerea*, this approach would provide valuable insights into the function of each effector and its contribution to disease development (Leisen et al., 2022). By employing marker-free transformation methods using CRISPR/Cas and telomere vectors recently developed in *B. cinerea* (Leisen et al., 2020), we could systematically dissect the role of these effectors in the virulence of *M. fructicola*. This would enhance our understanding of the molecular mechanisms underlying pathogenicity and aid in the development of targeted strategies for disease management. As part of future experiments, it is also planned to produce pure effectors related to virulence to elucidate plant receptors and further enhance our understanding of plant-pathogen interactions.

The use of purified effectors is very promising as a base for genetic analysis allowing us to explore the complexities of plant responses and uncover the underlying mechanisms involved. This approach facilitates the identification of genetic loci associated with susceptibility to the disease, offering valuable insights into how plants interact with pathogens. Such insights are crucial for developing effective strategies to enhance crop resilience. This perspective aligns with successful instances, such as the *P. nodorum*-wheat interaction, where strategically excluding effector-sensitive genotypes from breeding programs has contributed to bolstering the partial resistance of wheat to *P. nodorum* (Friesen et al., 2007; Liu et al., 2012; Shi et al., 2016; Downie et al., 2018; Cowger et al., 2020) Emphasizing the parallels between these strategies, our study encourages the adoption of refined breeding approaches that exclude susceptible genotypes, providing breeders and growers with a powerful tool to fortify crops against the targeted disease. The methodology developed in this study, enables large-scale trials to be conducted, testing various effectors in non-model plants. This advancement opens the door to assessing germplasm for differences in cell death induction, which may serve as an indicator of susceptibility to brown rot. By employing advanced techniques such as marker-assisted selection breeders can more precisely identify and eliminate susceptible genotypes, resulting in the development of crop varieties with enhanced resistance.

## Data Availability

The original contributions presented in the study are included in the article/**Supplementary Material**. Further inquiries can be directed to the corresponding authors.
