# Supplementary material for: Evaluation of cell death-inducing activity of Monilinia spp. effectors in several plants using a modified TRV expression system
Source: Front Plant Sci. 2024 Aug 16;15:1428613. doi: 10.3389/fpls.2024.1428613 (PMC11362074; doi:10.3389/fpls.2024.1428613)
Supplement: Supplementary file 1 [file DataSheet1.pdf]

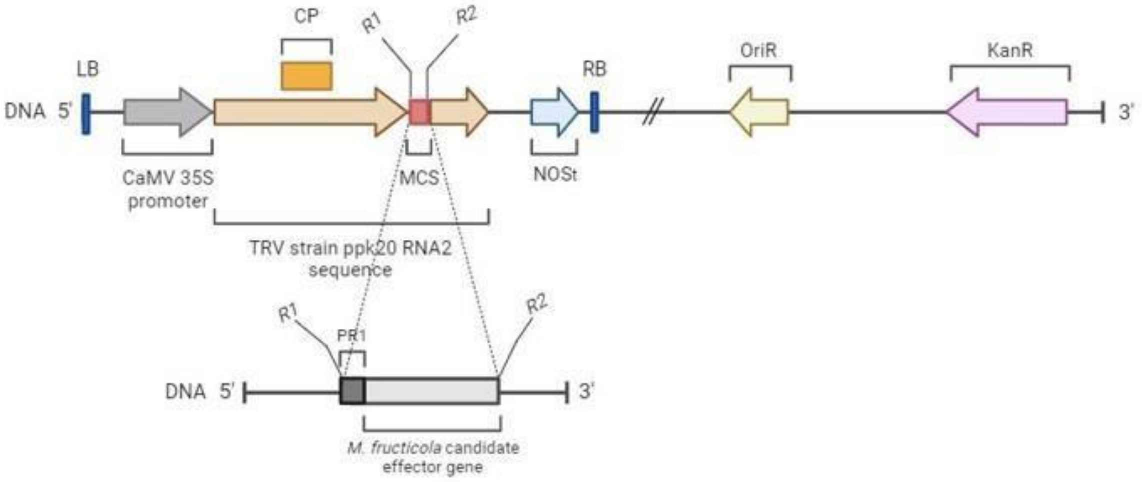

Supplementary Figure 1. Recombinant pTRV2 plasmid with *M. fructicola* genes. Schematic representation of the TRV2 plasmid (pYL156) and the construct that was cloned into it. The fragment was placed between the coat protein (CP) and the nopaline synthase terminator (NOSt) using restriction enzymes (R1 and R2). PR1, signal peptide of tobacco pathogene- sis-related protein PR1; MCS, multiple cloning site; LB and RB, left and right border of T-DNA; OriR, ColE1 origin of replication; KanR, kanamycin resistance gene.
