# Supplementary material for: Evaluation of cell death-inducing activity of Monilinia spp. effectors in several plants using a modified TRV expression system
Source: Front Plant Sci. 2024 Aug 16;15:1428613. doi: 10.3389/fpls.2024.1428613 (PMC11362074; doi:10.3389/fpls.2024.1428613)
Supplement: Supplementary file 2 [file DataSheet2.pdf]

TRV1+TRV2 (C-)

N. benthamiana | *S. lycopersicum* | *Prunus persica*

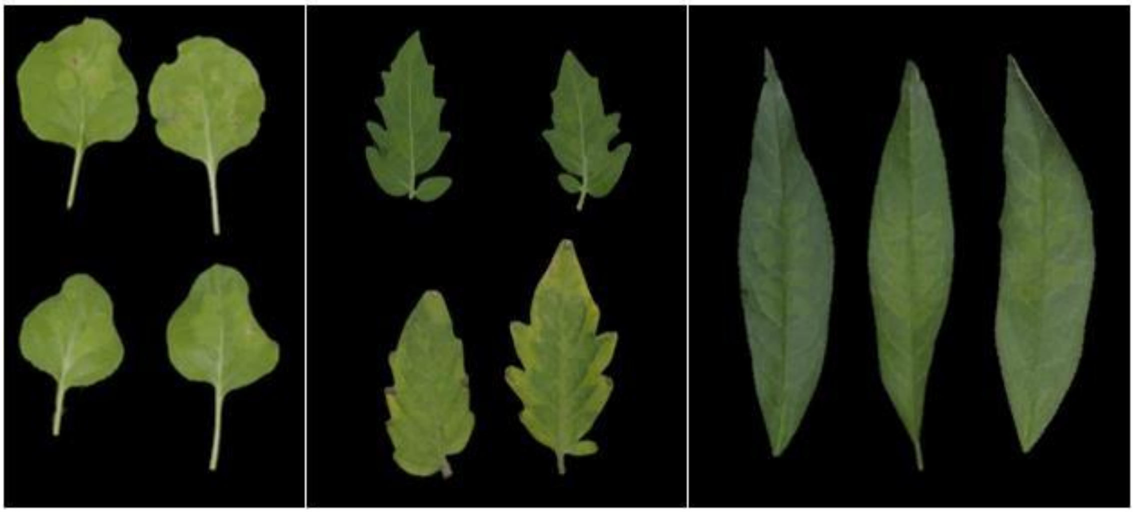

MFRU_002g05260

N. benthamiana | *S. lycopersicum* | *Prunus persica*

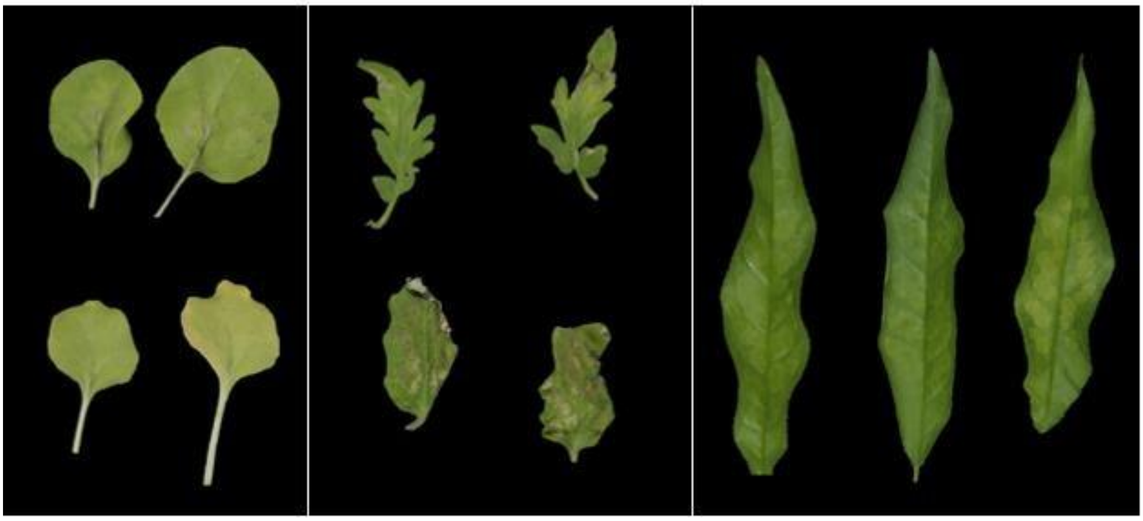

MFRU_004g00620

N. benthamiana | *S. lycopersicum* | *Prunus persica*

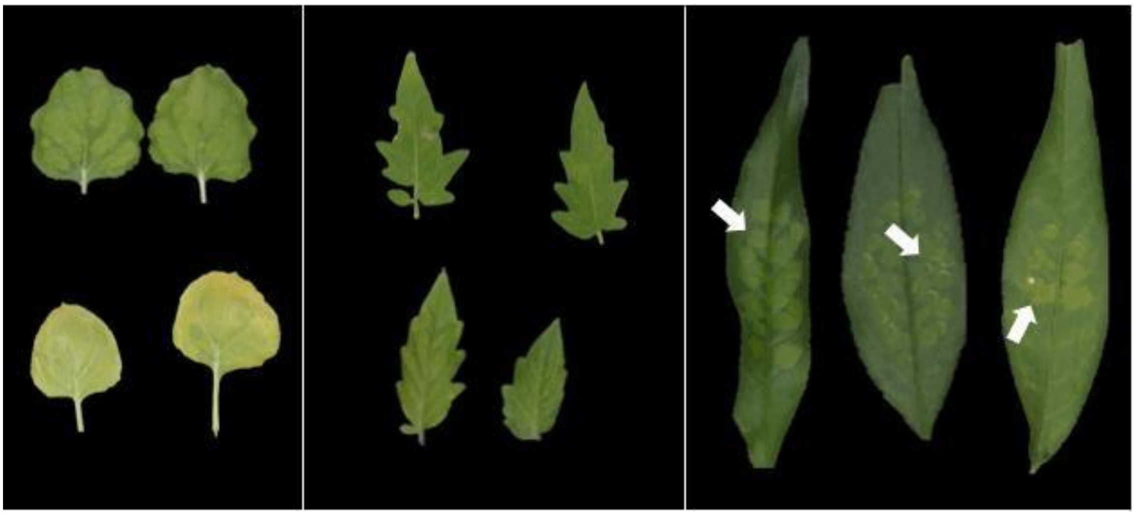

MFRU_004g02090

N. benthamiana *S. lycopersicum* *Prunus persica*

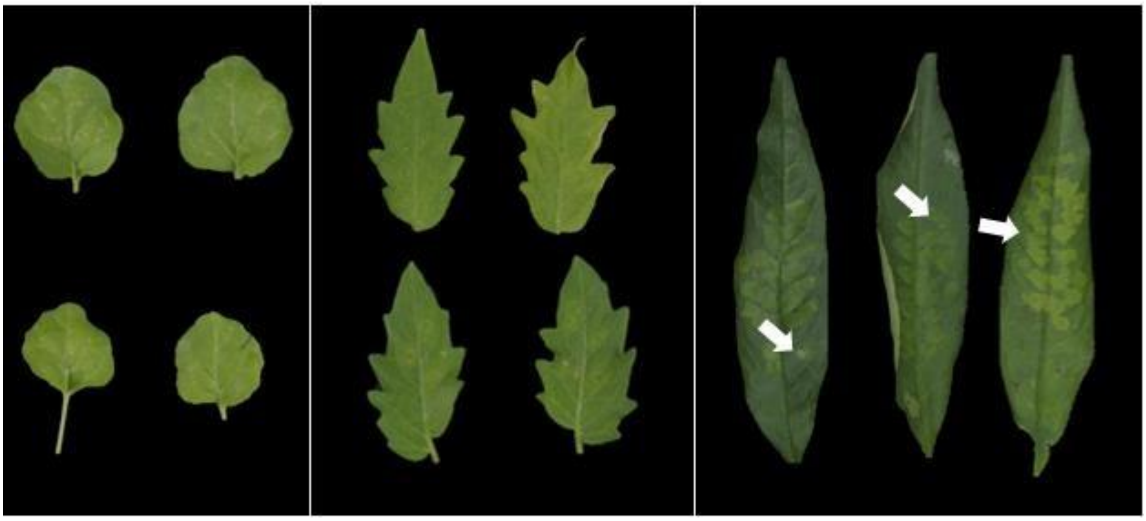

MFRU_004g02710

N. benthamiana *S. lycopersicum* *Prunus persica*

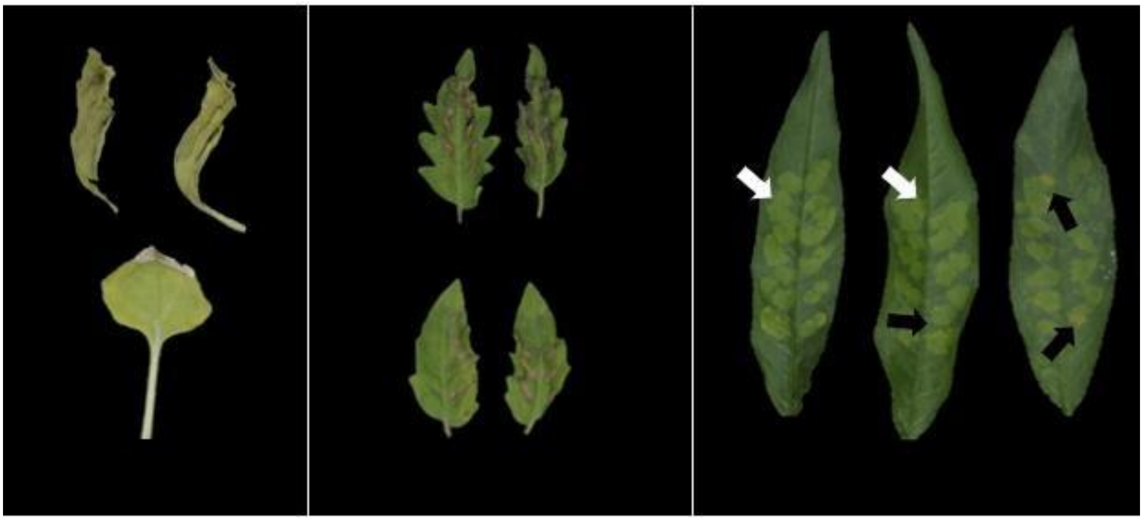

MFRU_004g02720

N. benthamiana *S. lycopersicum* *Prunus persica*

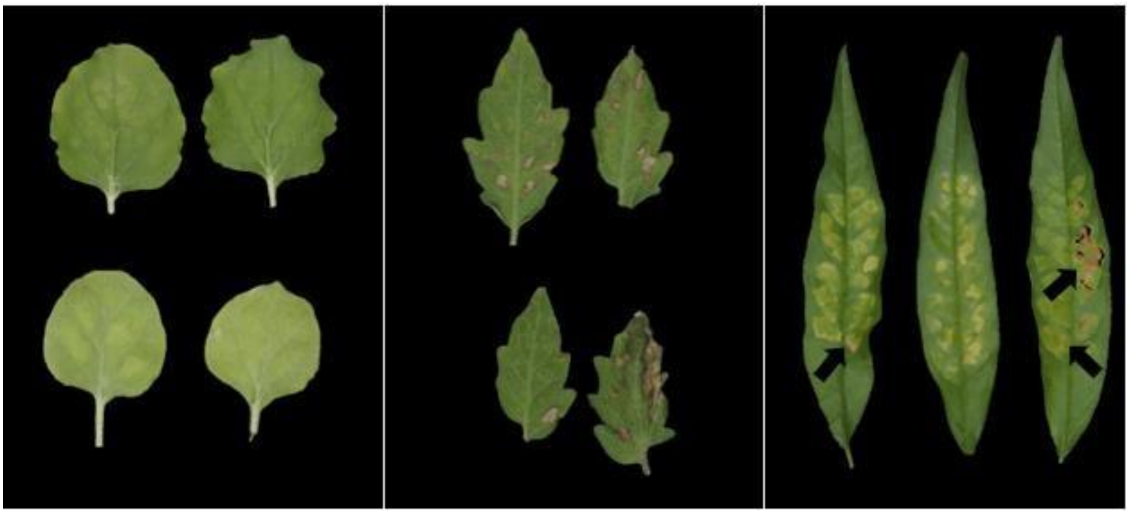

MFRU_005g0910

N. benthamiana | *S. lycopersicum* | *Prunus persica*

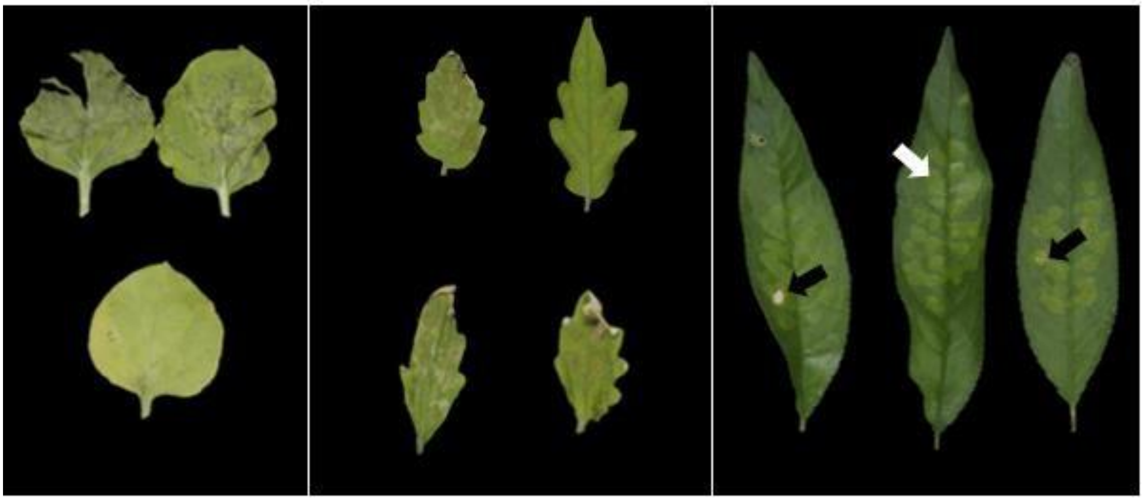

MFRU_008g03430

N. benthamiana | *S. lycopersicum* | *Prunus persica*

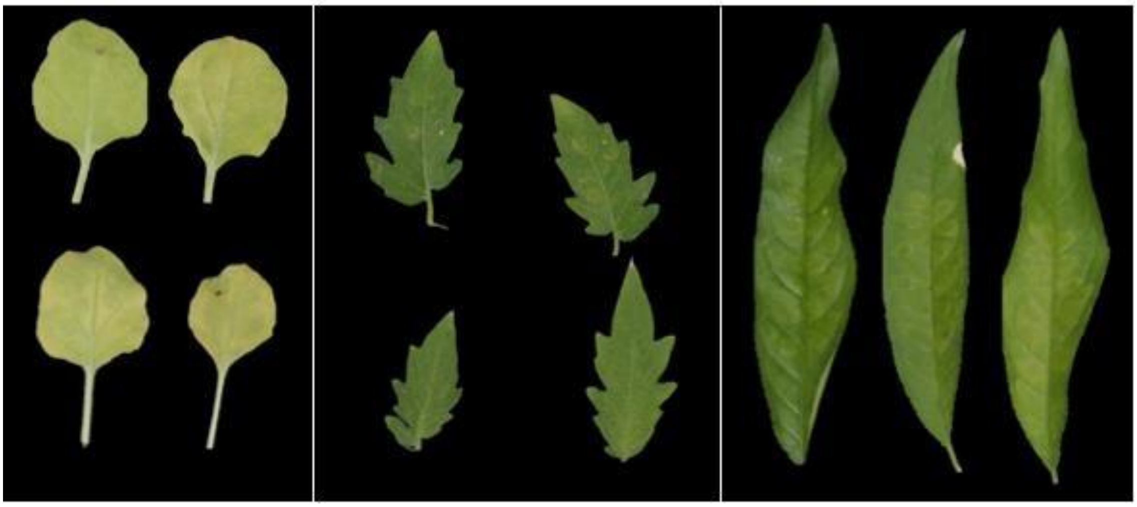

MFRU_010g02580

N. benthamiana | *S. lycopersicum* | *Prunus persica*

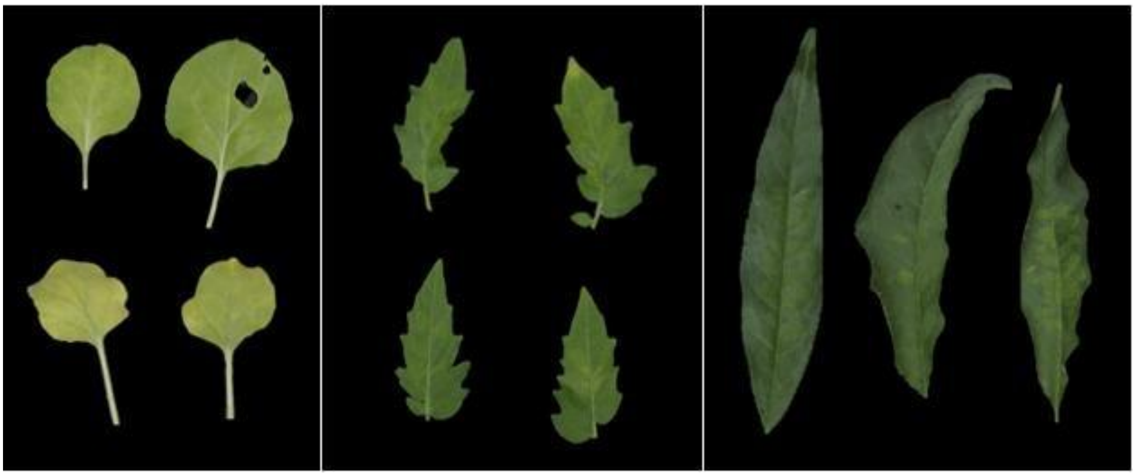

MFRU_014g02060

N. benthamiana | *S. lycopersicum* | *Prunus persica*

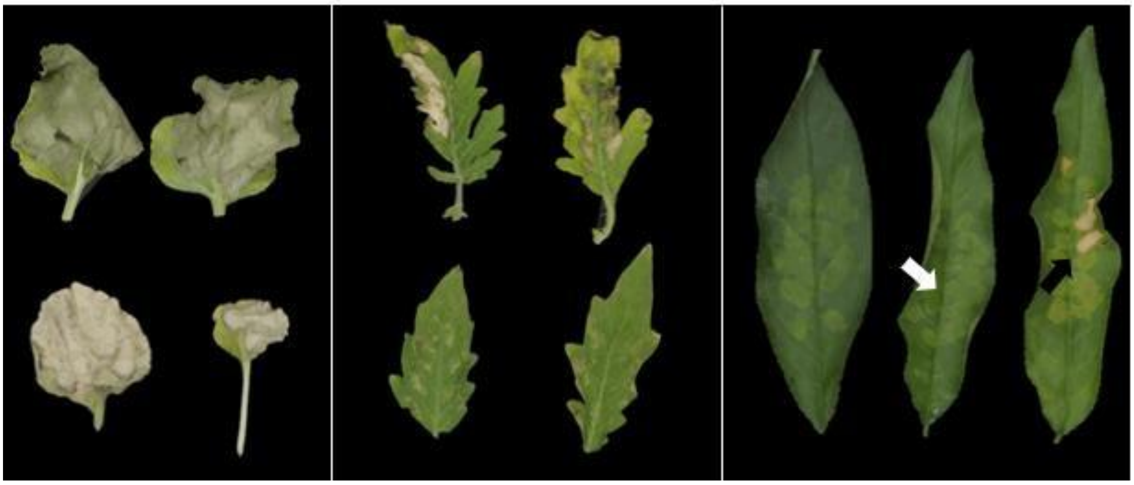

MFRU_018g01470

N. benthamiana | *S. lycopersicum* | *Prunus persica*

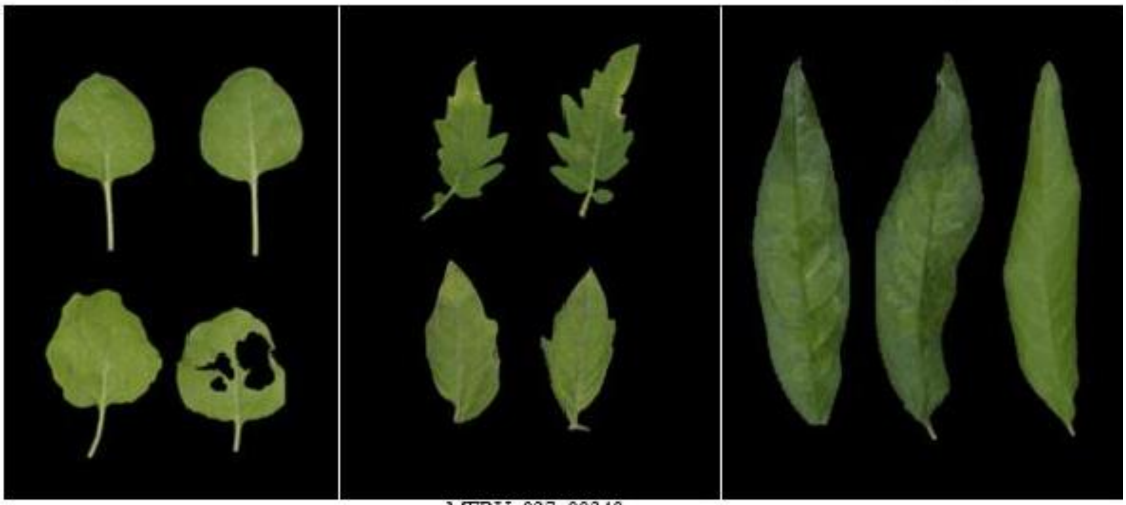

MFRU_027g00340

N. benthamiana | *S. lycopersicum* | *Prunus persica*

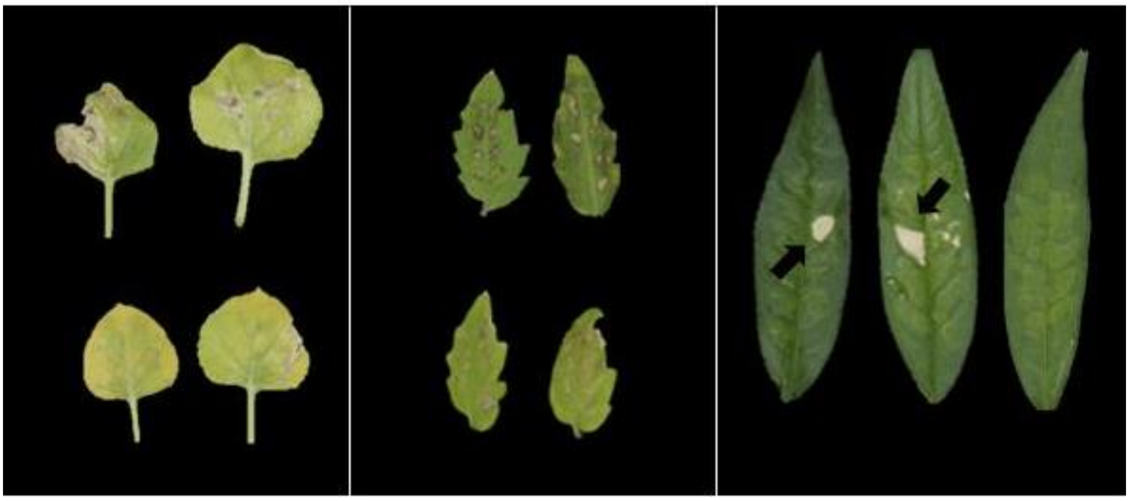

MFRU_030g00580

N. benthamiana | *S. lycopersicum* | *Prunus persica*

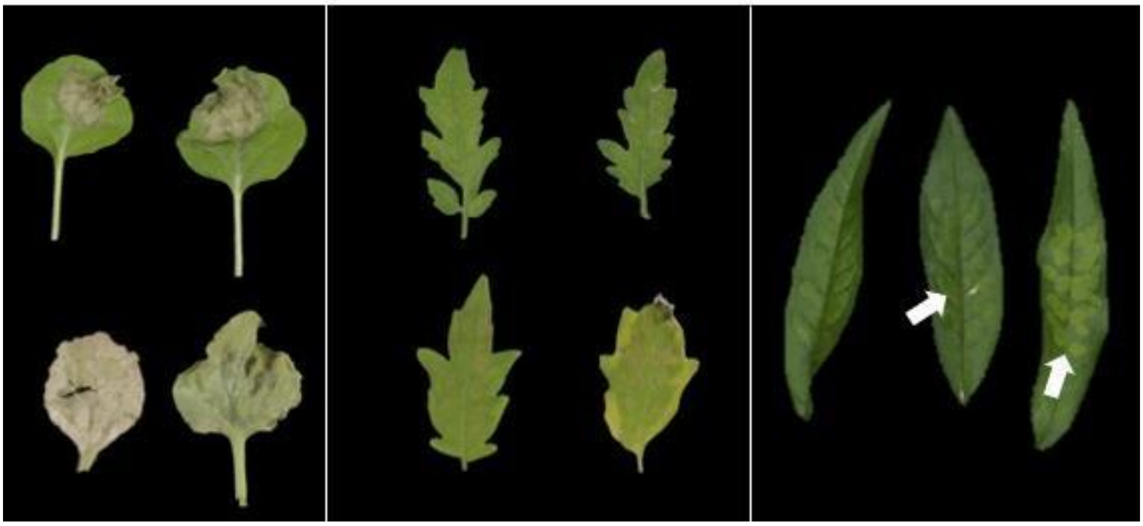

MFRU_030g00190

N. benthamiana | *S. lycopersicum* | *Prunus persica*

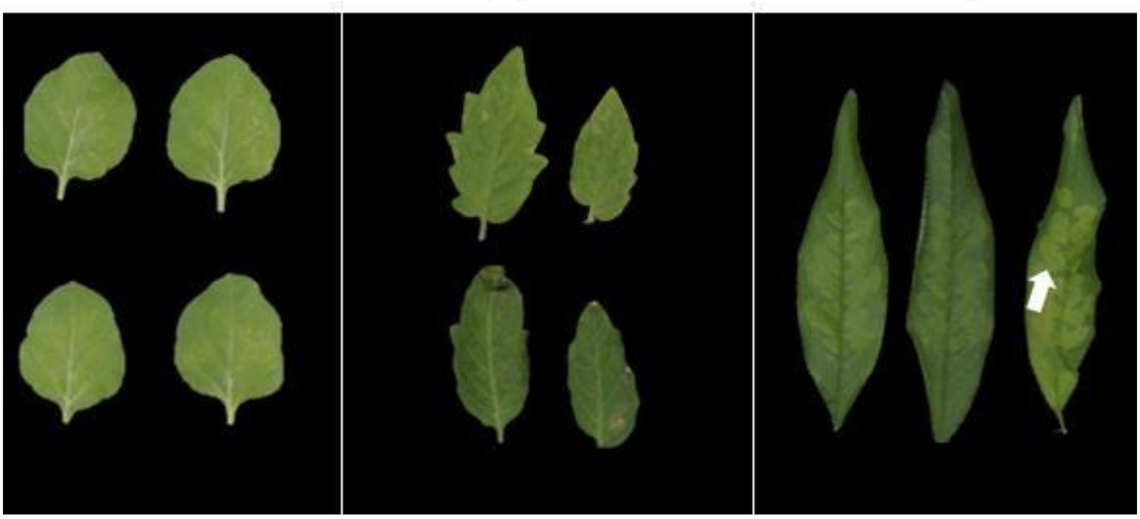

MFRU_048g00370

N. benthamiana | *S. lycopersicum* | *Prunus persica*

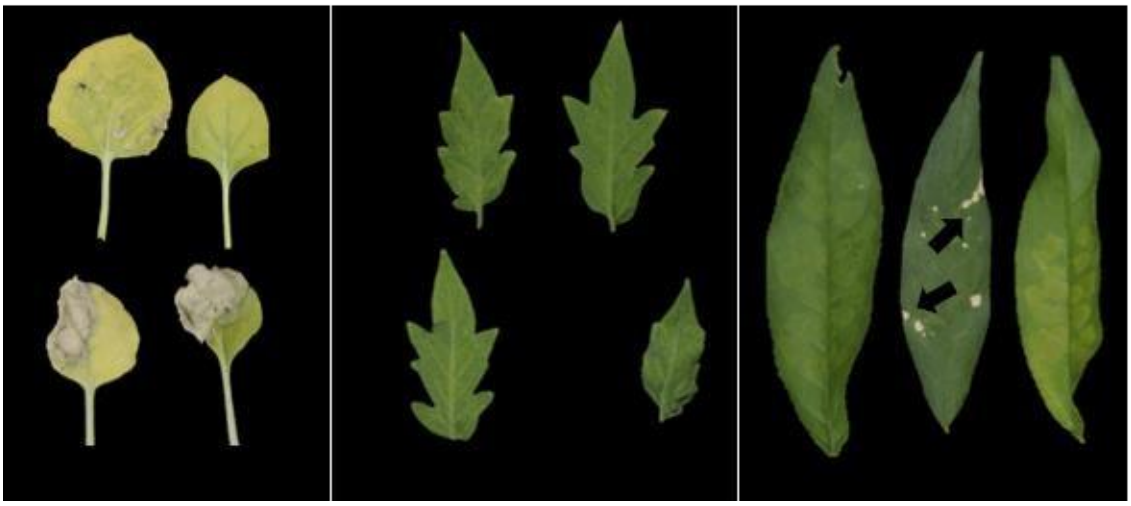

Supplementary Figure 2. *Agrobacterium tumefaciens* mediated-transient expression (ATTA) using modified TRV binary vector for expressing several *Monilinia fructicola* candidate effector proteins in *N. benthamiana* (first column), *S. lycopersicum* (middle column) and *Prunus persica* (third column). Black arrows highlight necrosis symptoms. White arrows highlight discoloration. First row of images shows the negative control (empty TRV2). Pictures were taken 7 days postinfiltration.
