# Supplementary material for: Evaluation of cell death-inducing activity of Monilinia spp. effectors in several plants using a modified TRV expression system
Source: Front Plant Sci. 2024 Aug 16;15:1428613. doi: 10.3389/fpls.2024.1428613 (PMC11362074; doi:10.3389/fpls.2024.1428613)
Supplement: Supplementary file 3 [file DataSheet3.pdf]

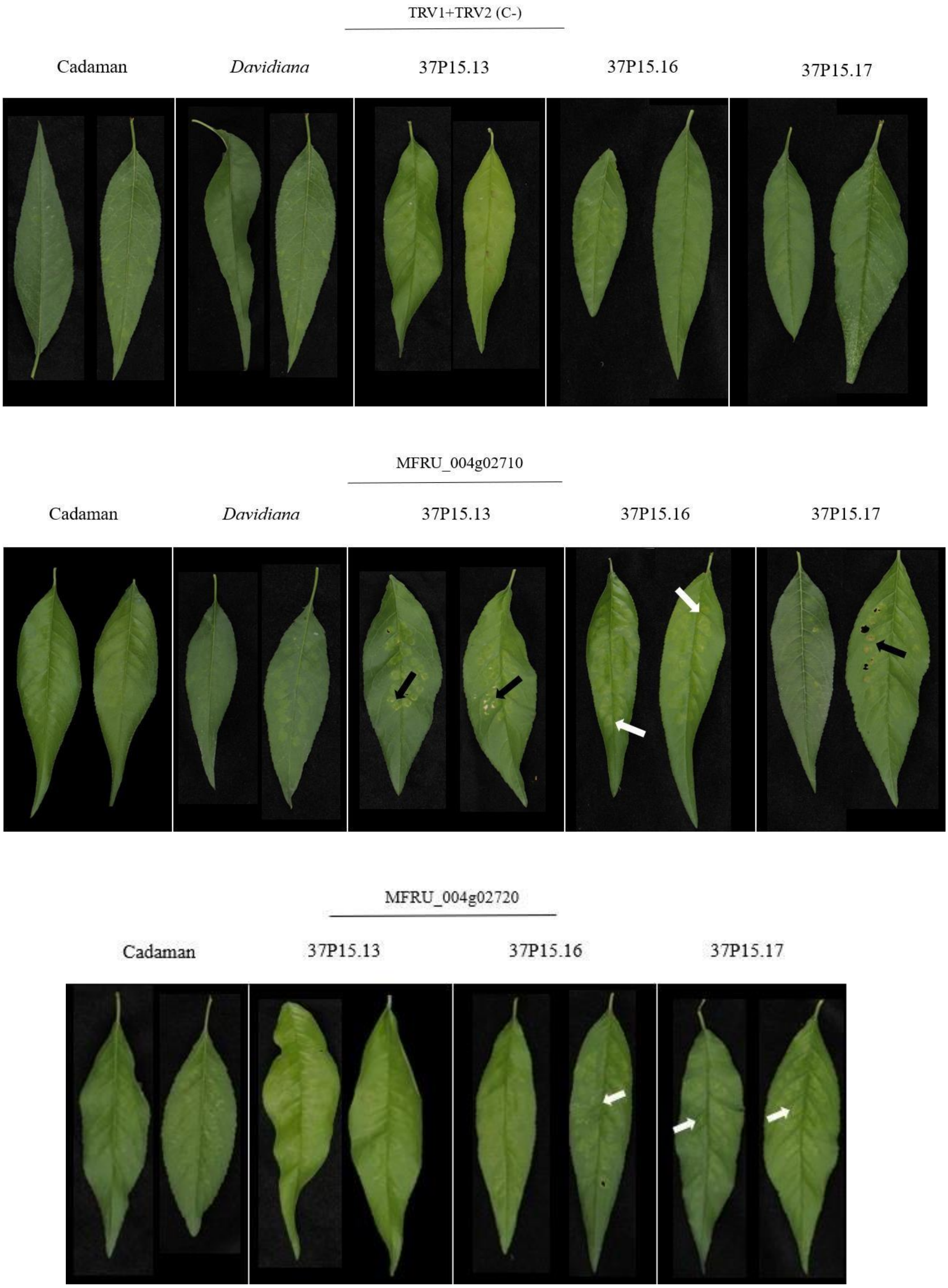
TRV1+TRV2 (C-)
Cadaman
Davidiana
37P15.13
37P15.16
37P15.17
MFRU_004g02710
Cadaman
Davidiana
37P15.13
37P15.16
37P15.17
MFRU_004g02720
Cadaman
37P15.13
37P15.16
37P15.17

MFRU_005g00910

Cadaman	*Davidiana*	37P15.13	37P15.16	37P15.17

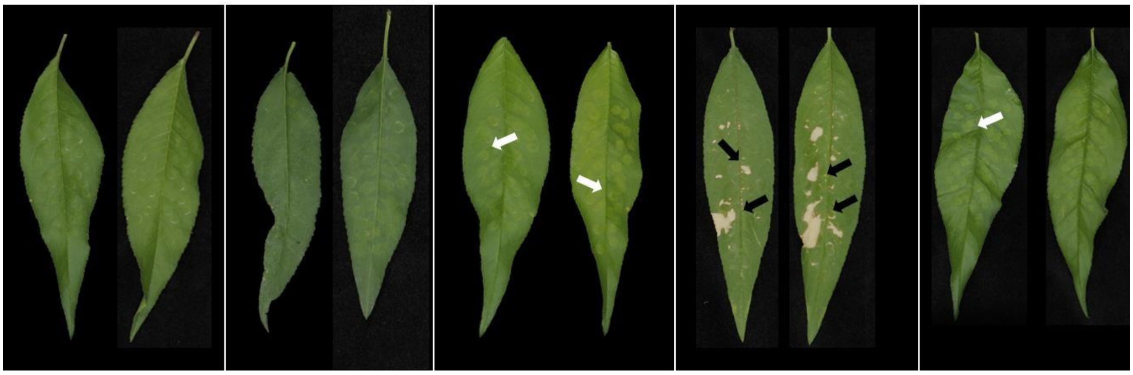

MFRU_014g02060

Cadaman	*Davidiana*	37P15.13	37P15.16	37P15.17

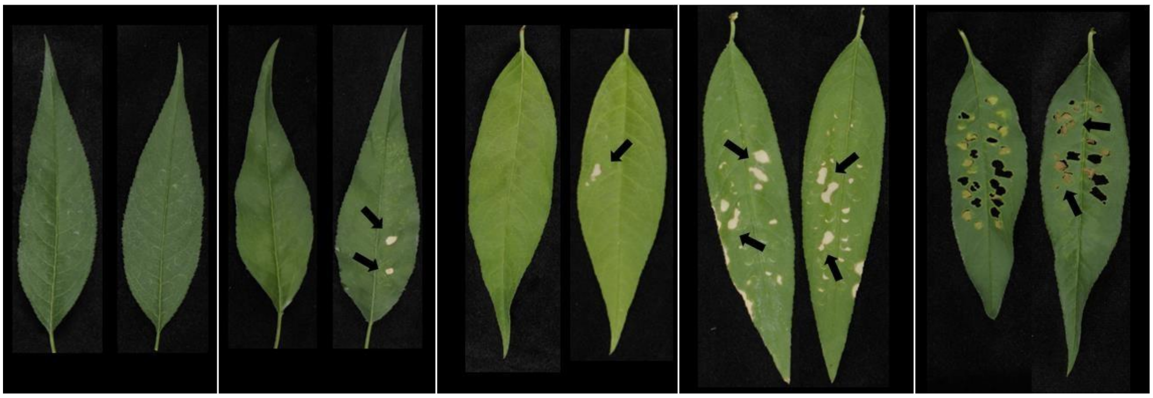

MFRU_027g00340

Cadaman	*Davidiana*	37P15.13	37P15.16	37P15.17

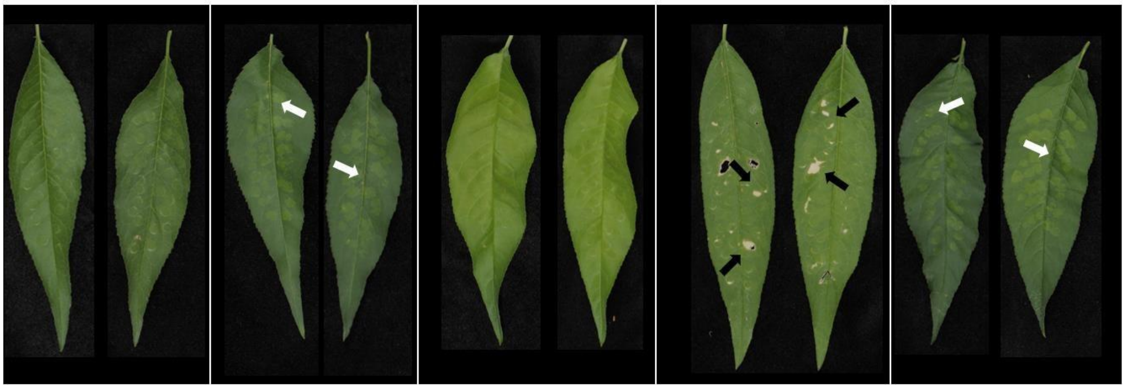

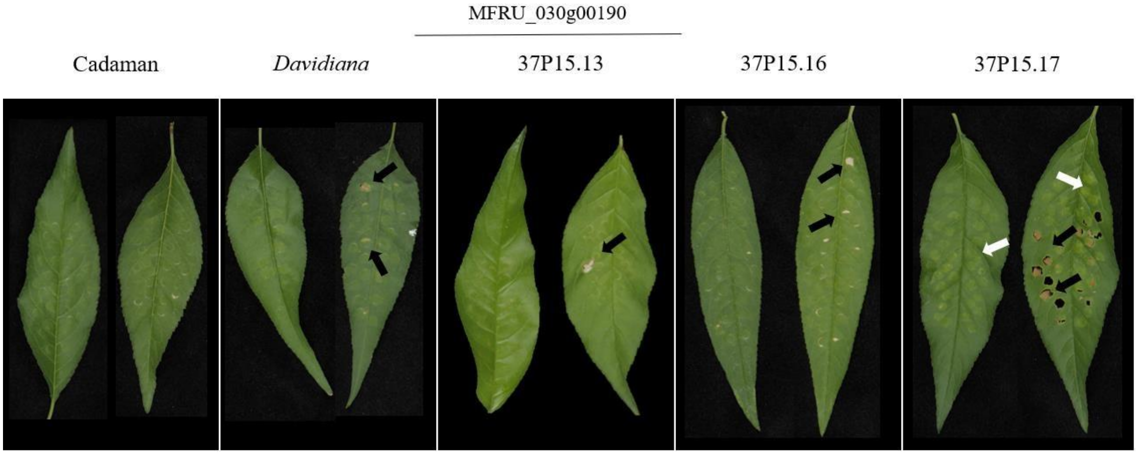

Supplementary Figure 3. *Agrobacterium tumefaciens* mediated-transient expression (ATTA) using modified TRV binary vector for expressing several *Monilinia fructicola* candidate effector proteins in different *Prunus* genotypes leaves: Cadaman (first row), *Davidiana* (second row), 37P15.13 (third row), 37P15.16 (fourth row) and 37P15.17 (last row). Black arrows highlight necrosis symptoms. White arrows highlight discoloration. First row of images is the negative control (empty TRV2). Pictures were taken 10 days postinfiltration.
